# Small‐Angle X‐Ray Scattering Studies of Block Copolymer Nano‐Objects: Formation of Ordered Phases in Concentrated Solution During Polymerization‐Induced Self‐Assembly

**DOI:** 10.1002/anie.202101851

**Published:** 2021-05-01

**Authors:** Matthew J. Rymaruk, Cate T. O'Brien, Csilla György, Bastien Darmau, James Jennings, Oleksandr O. Mykhaylyk, Steven P. Armes

**Affiliations:** ^1^ Dainton Building Department of Chemistry The University of Sheffield Sheffield S3 7HF UK; ^2^ Present address: Syngenta Jealott's Hill Bracknell Berkshire RG42 6EY UK

**Keywords:** block copolymers, lyotropic phases, polymerization-induced self-assembly, RAFT dispersion polymerization, SAXS

## Abstract

We report that polymerization‐induced self‐assembly (PISA) can be used to prepare lyotropic phases comprising diblock copolymer nano‐objects in non‐polar media. RAFT dispersion polymerization of benzyl methacrylate (BzMA) at 90 °C using a trithiocarbonate‐capped hydrogenated polybutadiene (PhBD) steric stabilizer block in n‐dodecane produces either spheres or worms that exhibit long‐range order at 40 % w/w solids. NMR studies enable calculation of instantaneous copolymer compositions for each phase during the BzMA polymerization. As the PBzMA chains grow longer when targeting PhBD_80_–PBzMA_40_, time‐resolved small‐angle X‐ray scattering reveals intermediate body‐centered cubic (BCC) and hexagonally close‐packed (HCP) sphere phases prior to formation of a final hexagonal cylinder phase (HEX). The HEX phase is lost on serial dilution and the aligned cylinders eventually form disordered flexible worms. The HEX phase undergoes an order–disorder transition on heating to 150 °C and a pure HCP phase forms on cooling to 20 °C.

## Graphical abstract

Polymerization‐induced self‐assembly (PISA) can produce lyotropic phases of block copolymer nano‐objects. Time‐resolved SAXS studies confirm that reversible addition–fragmentation chain transfer (RAFT) dispersion polymerization of benzyl methacrylate in *n*‐dodecane initially produces spheres exhibiting long‐range order that subsequently evolve to form hexagonally packed worms. Serial dilution leads to disordered worms.

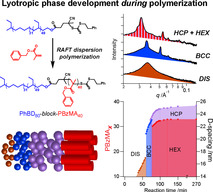

## Introduction

It is widely recognized that polymerization‐induced self‐assembly (PISA) is a powerful and versatile platform technology for the rational design of various types of block copolymer nano‐objects (e.g. spheres, worms, vesicles or lamellae).[^1^, ^2^, ^3^, ^4^] Moreover, PISA enables the efficient production of such nano‐objects in the form of concentrated dispersions,[^5^, ^6^, ^7^, ^8^] whereas traditional post‐polymerization processing routes are typically limited to dilute copolymer solutions.[^9^, ^10^, ^11^] In essence, PISA involves chain extension of a soluble homopolymer precursor using a suitable second monomer in an appropriate solvent. When the growing second block reaches a certain critical degree of polymerization it becomes insoluble, which drives in situ self‐assembly to form nascent nanoparticles.[^12^, ^13^] The polymerization continues within the monomer‐swollen nanoparticles, with the high local monomer concentration usually producing a rate acceleration that ensures high conversions (typically >95 %) within relatively short time scales.[^14^, ^15^] Thus, the monomer acts as a convenient processing aid or co‐solvent. In principle, the final diblock copolymer morphology is governed solely by the relative volume fractions of the soluble and insoluble blocks, as indicated by the geometric packing parameter.[^11^, ^16^] In practice, kinetically trapped spheres can be observed under various reaction conditions.[^17^, ^18^, ^19^, ^20^] To ensure access to well‐defined worms or vesicles, the insoluble structure‐directing block should be relatively long compared to the soluble steric stabilizer block.[^3^, ^4^, ^11^, ^21^] We, and others, have demonstrated that the construction of pseudo‐phase diagrams is extremely useful for identifying appropriate PISA formulations for the reproducible production of pure copolymer morphologies.[^5^, ^15^, ^22^] This systematic approach is particularly important when targeting diblock copolymer worms because this elusive morphology typically occupies relatively narrow phase space.[^23^, ^24^] As a result, robust design rules are well established for various PISA formulations based on dispersion polymerization.[^2^, ^3^]

Block copolymer worms offer potential applications as thickeners for a wide range of solvents, including water,[^25^, ^26^] polar solvents such as alcohol,[^27^, ^28^] and non‐polar solvents such as *n*‐alkanes, mineral oil or silicone oil.[^14^, ^15^, ^29^, ^30^] Moreover, semi‐concentrated worm dispersions form relatively soft, free‐standing gels at ambient temperature owing to multiple inter‐worm contacts.^[31]^ Rheological studies confirm that such gels are highly sensitive to shear‐induced flow,[^32^, ^33^] which enables their injection using a syringe. Certain aqueous worm gels exhibit thermoresponsive behavior; on cooling to around 5 °C, they undergo a reversible worm‐to‐sphere transition that causes in situ degelation.^[25]^ In contrast, worm gels prepared in organic solvents undergo degelation on heating as a result of the same morphological transition.[^14^, ^28^, ^29^, ^30^] In both cases, this behavior can be explained in terms of surface plasticization of the worm cores, which leads to a subtle reduction in the effective packing parameter.[^16^, ^34^, ^35^]

Relatively dilute diblock copolymer worm dispersions have been studied for several decades.[^9^, ^10^, ^23^, ^36^, ^37^, ^38^, ^39^] Similarly, the self‐assembly of block copolymers in the solid state[^40^, ^41^] has been exploited for numerous applications, including the production of synthetic rubber,^[42]^ the design of nanoporous membranes to aid water purification,^[43]^ and polymer electrolytes for batteries or fuel cells.[^44^, ^45^] It is also well‐known that concentrated dispersions of diblock copolymer nano‐objects can form lyotropic phases that exhibit long‐range order.[^46^, ^47^] For example, in their seminal study of block copolymer worms in aqueous solution, Bates and co‐workers reported that a near‐symmetrical poly(ethylene oxide)‐poly(butadiene) (PEO–PBD) diblock copolymer formed hexagonally packed cylinders at or above 10 % w/w copolymer concentration.^[36]^ Similarly, Lodge and co‐workers reported the lyotropic self‐assembly of diblock copolymers in the presence of various selective non‐polar solvents.[^46^, ^48^] In particular, many phases can be accessed for polyisoprene–polystyrene (PI–PS) diblock copolymers depending on the copolymer concentration and the solvent quality.^[48]^ However, these nanostructured solvent‐swollen phases are traditionally produced by post‐polymerization processing, which is normally conducted in relatively dilute solution using a VOC as a co‐solvent.

In contrast, Zhang et al. reported the PISA synthesis of concentrated diblock copolymer worms via the RAFT dispersion polymerization of a cholesterol‐based (meth)acrylic monomer in an ethanol/water mixture.^[49]^ Electron microscopy studies indicated that the cholesterol units formed a smectic phase within the worm cores, which stabilized this anisotropic morphology over a relatively wide range of copolymer compositions. Moreover, small‐angle X‐ray scattering (SAXS) provided evidence for the “apparent preferential order” of neighboring worms. However, this was assumed to be a sample preparation artifact rather than a lyotropic phase formed during PISA. More recently, An and co‐workers reported the formation of bicontinuous mesophases (“cubosomes”) within diblock copolymer microparticles during RAFT dispersion alternating copolymerization of styrene with pentafluorostyrene in ethanol.[^50^, ^51^] The addition of toluene as a plasticizer was required to access these phases, which were obtained by targeting relatively long structure‐directing insoluble blocks.

As far as we are aware, the formation of bulk lyotropic phases of block copolymer worms during their PISA synthesis has not been previously observed. Herein we report the PISA synthesis of highly concentrated dispersions of diblock copolymer worms that form a hexagonally packed cylinder (HEX) phase when prepared directly in *n*‐dodecane. We use SAXS to study (i) the in situ evolution in copolymer morphology and intermediate lyotropic phases that occur during PISA, (ii) the serial dilution that eventually leads to a conventional dispersion of worm‐like micelles, and (iii) the effect of thermal annealing on these lyotropic phases.

## Results and Discussion

**Figure 1:**
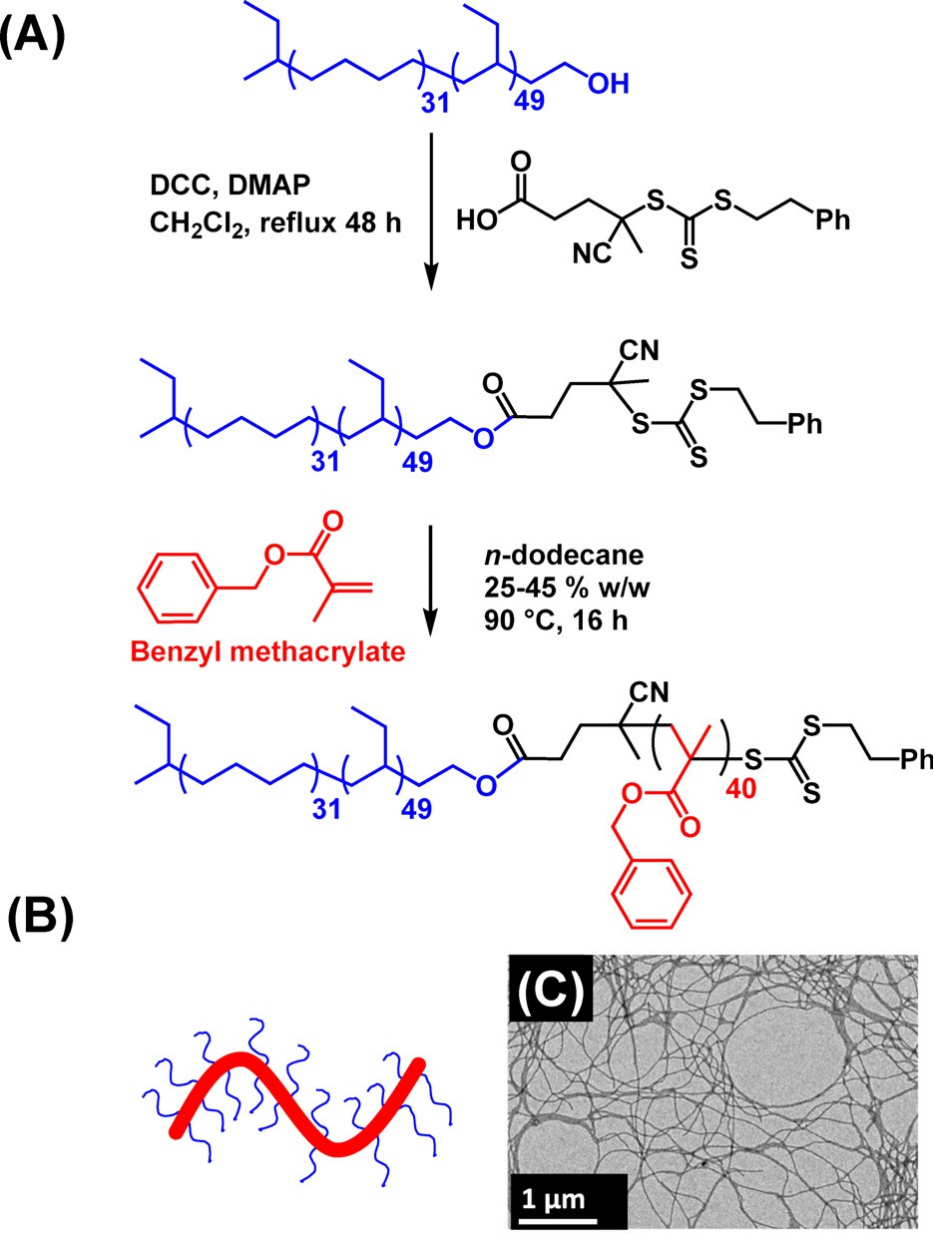
A) Chemical structure of the PhBD_80_ macro‐RAFT agent (blue) and its chain extension with BzMA (red) to form a PhBD_80_–PBzMA_40_ diblock copolymer via PISA at 90 °C. B) Schematic representation of PhBD_80_–PBzMA_40_ worms, with a PhBD_80_ stabilizer block and a PBzMA_40_ core‐forming block. C) Representative TEM image recorded after drying a dilute dispersion of PhBD_80_–PBzMA_40_ worms prepared after serial dilution of the as‐synthesized 40 % w/w dispersion at 20 °C.

Recently, we reported a pseudo‐phase diagram for PhBD_80_‐PBzMA_x_ nano‐objects that enables the reproducible PISA synthesis of well‐defined spheres, worms and vesicles in *n*‐dodecane.^[52]^ Interestingly, the formation of a pure worm phase required such PISA syntheses to be conducted at 40 % w/w solids, with lower copolymer concentrations merely producing kinetically trapped spheres. Presumably, this is because the stochastic 1D fusion of multiple spheres that is required to form worms is not favored under more dilute conditions. As far as we are aware, this constitutes the most concentrated worm dispersion yet reported for any PISA formulation conducted in non‐polar media.^[49]^ More specifically, a PhBD_80_–PBzMA_40_ dispersion prepared at 40 % w/w solids formed a stiff, transparent gel at 20 °C and a highly anisotropic worm morphology was confirmed by TEM studies of the serially diluted dispersion (see Figure 1).

**In situ SAXS analysis**. In our earlier report,^[52]^ these block copolymer nano‐objects were characterized using GPC, TEM, DLS and rheology. However, no SAXS studies were undertaken. In the present study, the evolution of copolymer morphology during PISA was monitored using time‐resolved SAXS when targeting PhBD_80_‐PBzMA_40_ worms at 40 % w/w solids. We and others have previously studied the in situ evolution in copolymer morphology for various PISA formulations.[^53^, ^54^, ^55^, ^56^] This approach enables the onset of micellization to be identified, as well as the observation of sphere‐to‐worm and worm‐to‐vesicle transitions during the polymerization.^[50]^

**Figure 2:**
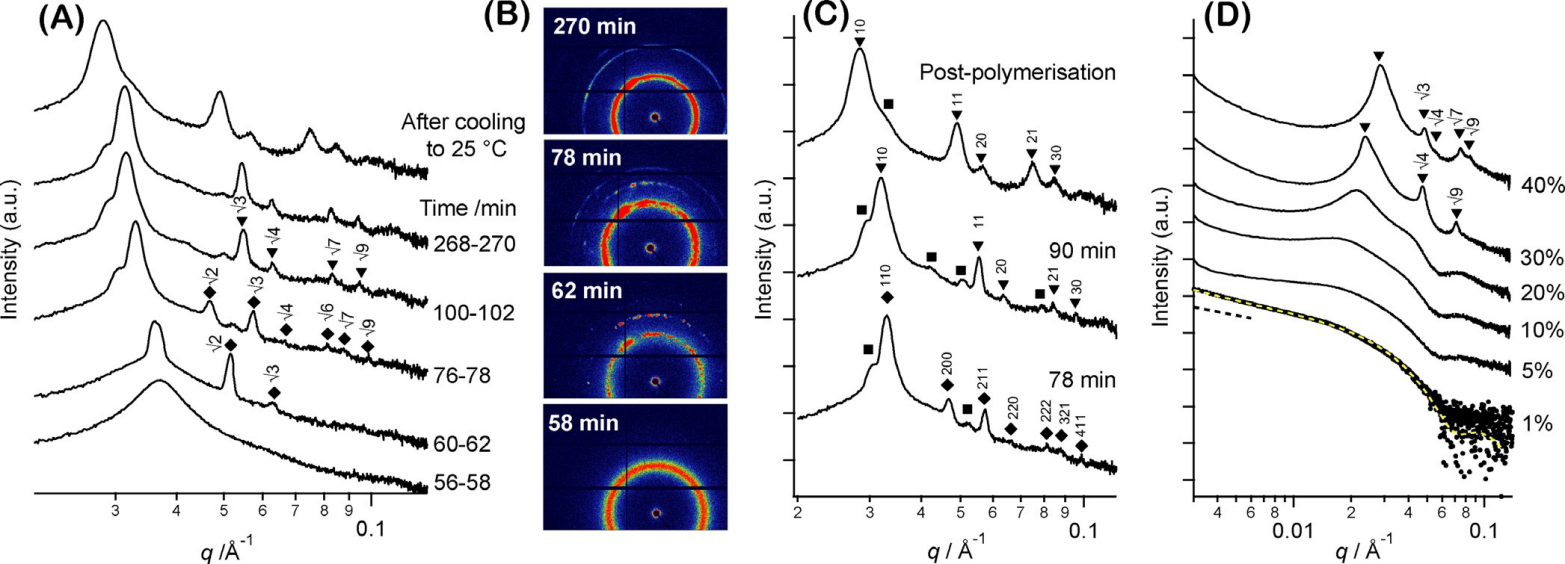
Selected A) 1D and B) 2D SAXS patterns recorded during the PISA synthesis of PhBD_80_–PBzMA_40_ diblock copolymer nano‐objects at 40 % w/w solids in *n*‐dodecane. Frames were continuously collected using a 2 min exposure time per frame for 270 min (a total of 135 frames), so reaction times are expressed as ranges in (A). The artifactual horizontal and vertical black stripes observed in the 2D SAXS patterns shown in (B) correspond to dead areas of the 1 M Pilatus detector. C) Representative indexed patterns: (bottom) body‐centered cubic (BCC) phase observed after 78 min with corresponding diffraction peaks indicated by diamonds and labeled using Miller indices, *hkl*; (middle) hexagonal cylinder (HEX) phase formed after 90 min with corresponding diffraction peaks indicated by triangles and labeled by Miller indices, *hk*; (top) HEX phase observed at the end of the BzMA polymerization after cooling to 25 °C for 5 h. Peak positions of unknown phase(s) are indicated by squares (see main text and supporting information for their eventual assignment). D) SAXS patterns recorded at 21 °C for PhBD_80_–PBzMA_40_ worms synthesized at 40 % w/w solids after serial dilution to 30, 20, 10, 5.0 or 1.0 % w/w in *n*‐dodecane. The data fit (see yellow dashed line) obtained for the SAXS pattern recorded at 1.0 % w/w was obtained using a worm‐like micelle model reported in the literature.^[60]^ The dashed black line represents a slope of −1 that is characteristic of worm‐like nano‐objects.^[26]^ 1D SAXS patterns are offset by an arbitrary multiplication factor to avoid overlap of the data.

Inspecting Figure 2 A,B), several stages of structural organization could be identified from the significant changes observed in the SAXS patterns as the BzMA polymerization progressed. Relatively featureless patterns were recorded within the first 28 min of the reaction, suggesting the presence of soluble copolymer chains and/or the formation of relatively loose, ill‐defined aggregates. Unlike in situ SAXS studies of more dilute formulations in which well‐defined local minima signified the formation of nano‐objects,[^53^, ^54^, ^55^] there was no evidence for any particle form factor. Instead, only a broad structure peak was detected (Figure 2 A). This feature became increasingly intense, and the peak maximum gradually shifted to lower *q* over the first 58 min of the polymerization (Figure S3).

A remarkable change in the 1D SAXS pattern was observed after 1 h, whereby three sharp Bragg peaks (principal scattering peak, q*=0.037 Å^−1^) emerged from the initial broad structure peak, with higher‐order peaks located at *q*/q*=$\sqrt{2}$ and $\sqrt{3}$ (Figure 2 A, see pattern recorded at 60–62 min). The corresponding 2D SAXS pattern observed at 62 min (Figure 2 B) shows a series of diffraction spots, commonly associated with reflections from crystallographic planes of large domains formed by spheres exhibiting long‐range order.^[57]^ After 76 min, additional peaks appeared at *q*/q*=$\sqrt{4}$ , $\sqrt{6}$ , $\sqrt{7}$ and $\sqrt{9}$ that grew in intensity as the polymerization progressed (Figure 2 A, see 78 min). The relative peak positions and their associated intensities are consistent with a body‐centered cubic (BCC) unit cell, in which Miller indices 110, 200, 211, 220, 222, 321 and 411 respectively can be assigned to the observed peaks (see Figure 2 C for a full assignment).^[58]^ Such a BCC structure is usually formed by spherical particles.^[59]^ This is because at this relatively early stage of the polymerization, where the volume fraction of the growing PBzMA block is much lower than that of the PhBD_80_ block, the diblock copolymer chains should self‐assemble to form spherical micelles.^[53]^

After 78 min, various additional peaks (see square symbols in Figure 2 C) are observed that cannot be assigned to the same BCC phase. The corresponding 2D scattering pattern (Figure 2 B) revealed an additional series of diffraction spots located at around 0.03 Å^−1^, which suggests the coexistence of BCC with a second phase(s). As the BzMA polymerization continues, the 200 peak of the BCC phase is no longer observed after 90 min (Figure 2 C). Moreover, after this time point the three most intense peaks are located at *q*/q*=1, $\sqrt{3}$ and $\sqrt{4}$ , while weaker peaks are located at *q*/q*=$\sqrt{7}$ , $\sqrt{9}$ (Figure 2 A, see 100–102 min); this is consistent with a hexagonally packed cylinder (HEX) phase.^[48]^ As the BzMA polymerization proceeds to completion, the two diffraction rings with evenly distributed intensities observed at approximately 0.03 Å^−1^ and 0.05 Å^−1^ in the 2D SAXS patterns (Figure 2 B, 270 min) indicate isotropic (no large domains and/or preferable orientation) structure and are consistent with a hexagonal phase (Figure 2 A, see 268–270 min). The unidentified peak observed at lower *q* is associated with diffraction spots most likely produced by large crystallite domains, suggesting another coexisting phase. At the end of polymerization, the most intense scattering peaks are assigned to the HEX phase (Figure 2 A, see 268–270 min).

After cooling the 40 % w/w copolymer dispersion to 25 °C for 5 h, most of these features could still be observed, suggesting the persistence of this ordered phase as the dominant structure (Figure 2 C, top). This cylinder phase comprises locally aligned worms that possess significantly fewer degrees of freedom than the more dilute dispersions of randomly oriented, non‐interacting worms that have often been reported for PISA syntheses conducted in non‐polar media.[^15^, ^29^, ^33^, ^53^, ^61^] To the best of our knowledge, this is the first time that any long‐range order has been observed for block copolymer nano‐objects during their PISA synthesis.

**Lyotropic phase behavior**. When the as‐synthesized PhBD_80_–PBzMA_40_ dispersion was diluted to a sufficiently low concentration for TEM analysis, well‐defined worm‐like micelles were observed (Figure 1 C). This loss of long‐range order upon dilution was examined in more detail via sequential dilution of the copolymer dispersion from 40 % w/w to 30, 20, 10, 5 or 1 % w/w using *n*‐dodecane prior to performing SAXS measurements at 21 °C (Figure 2 D).

Dilution to 30 % w/w resulted in a significant increase in domain spacing, as evidenced by a shift in the primary scattering peak to lower *q*. In addition, the $\sqrt{3}$ and $\sqrt{7}$ peaks disappeared, leaving only higher‐order peaks corresponding to *q*/q*=$\sqrt{4}$ and $\sqrt{9}$ (Figure 2 D). On dilution, the cylinders should become more loosely packed, which results in the HEX phase losing its six‐fold rotation axis symmetry to form stacked layers of cylinders producing first, second and third order reflections (10, 20 and 30, respectively). Moreover, the greater separation distance between neighboring cylinders could result in the disappearance of the $\sqrt{7}$ peak if its position coincides with the first minimum of the cylinder (worm) form factor observed for more dilute dispersions (Figure 2 D). The emergence of a broad feature beneath the primary scattering peak indicates a significant proportion of disordered worms that lack any long‐range hexagonal packing. Further dilution resulted in the complete loss of all sharp Bragg peaks. For example, just two very broad structure peaks were observed at a copolymer concentration of 20 % w/w, suggesting only rather weak correlation between neighboring particles at this copolymer concentration. These structure peaks disappeared on further dilution: only a local minimum at *q*≈0.064 Å^−1^ (corresponding to the particle form factor) was discernible at a copolymer concentration of 5.0 % w/w, which is consistent with isolated, non‐interacting worms.[^26^, ^29^, ^61^] Furthermore, the SAXS pattern corresponding to the lowest copolymer concentration (1.0 % w/w) exhibited a low *q* gradient of approximately −1 and could be satisfactorily fitted using a worm‐like micelle model^[60]^ (see Figure 2 D).

SAXS analysis of a 1.0 % w/w dispersion of PhBD_80_‐PBzMA_40_ worms in *n*‐dodecane indicated a mean cross‐sectional diameter of around 19 nm for the worm cores. This is in reasonably good agreement with the mean worm width of 22±4 nm estimated by digital image analysis of electron micrographs recorded after drying a dilute copolymer worm dispersion (see Figure 1 C).

Thus, these results suggest that serial dilution of the original 40 % w/w PhBD_80_–PBzMA_40_ dispersion using a selective solvent for the PhBD_80_ block (*n*‐dodecane) leads to transformation of the hexagonally packed cylinder phase into a disordered worm phase. This is consistent with the lyotropic behavior typically exhibited by diblock copolymers in the presence of a selective solvent.[^48^, ^62^] It is perhaps noteworthy that the chemical structure of the PhBD_80_–PBzMA_40_ diblock copolymer examined herein bears some resemblance to that of PI–PS[^57^, ^63^] and poly(ethylene‐butylene)–polystyrene (EBS/SEBS)[^64^, ^65^] diblock copolymers, whose lyotropic self‐assembly behavior has been extensively investigated. For these latter two systems, both micellar cubic phases and hexagonal phases have often been observed under various conditions.[^65^, ^66^]

To complete this series of measurements on the concentration‐dependent self‐assembly behavior, the solid‐state morphology of the final PhBD_80_–PBzMA_40_ diblock copolymer was also assessed. After isolating PhBD_80_–PBzMA_40_ from *n*‐dodecane by precipitation into excess ethanol, the resulting dry copolymer powder was analyzed by SAXS over a wide temperature range (Figure S4). Only broad, low intensity peaks were observed at 40 °C, which suggests weak segregation within a relatively disordered non‐equilibrium phase that is formed following precipitation.[^67^, ^68^] However, thermal annealing drives further microphase separation, which leads to the appearance of several sharp peaks at higher *q*. On heating above 100 °C, at least three equally spaced peaks are observed at *q*/q*=1, 2 and 3, indicating the formation of a lamellar phase.[^46^, ^48^] This is consistent with the near‐symmetrical structure of this PhBD_80_–PBzMA_40_ diblock copolymer: it has a PhBD volume fraction of 0.45 for which a lamellar morphology would be expected in the solid state.^[41]^ Thus, solvation of the PhBD stabilizer chains by *n*‐dodecane increases the effective volume fraction of this block relative to that of the non‐solvated PBzMA block.^[53]^ Such selective swelling switches the preferred morphology from lamellae to close‐packed cylinders (or spheres) comprising PBzMA cores.[^16^, ^35^]

The domain spacing, *L*_0_, for the lamellar phase formed by the PhBD_80_–PBzMA_40_ diblock copolymer in the solid state can be calculated using the relation *L*_0_=2π/q*. This parameter ranged from 16.8 nm at 108 °C (the temperature at which a well‐defined lamellar phase was first observed during thermal annealing) to 14.7 nm at 280 °C. Despite the relatively low molecular weight of this diblock copolymer, its order–disorder transition (ODT) temperature is unusually high and could not be observed under the experimental conditions used in the present study (i.e., it must exceed 280 °C, Figure S4). Such observations suggest that this is a high *χ* diblock copolymer system that should enable the construction of nanostructured materials comprising sub‐10 nm domain spacings.^[69]^ In this context, we note that structurally similar PI–PS diblock copolymers also possess a relatively high *χ* value.^[63]^

**Figure 3:**
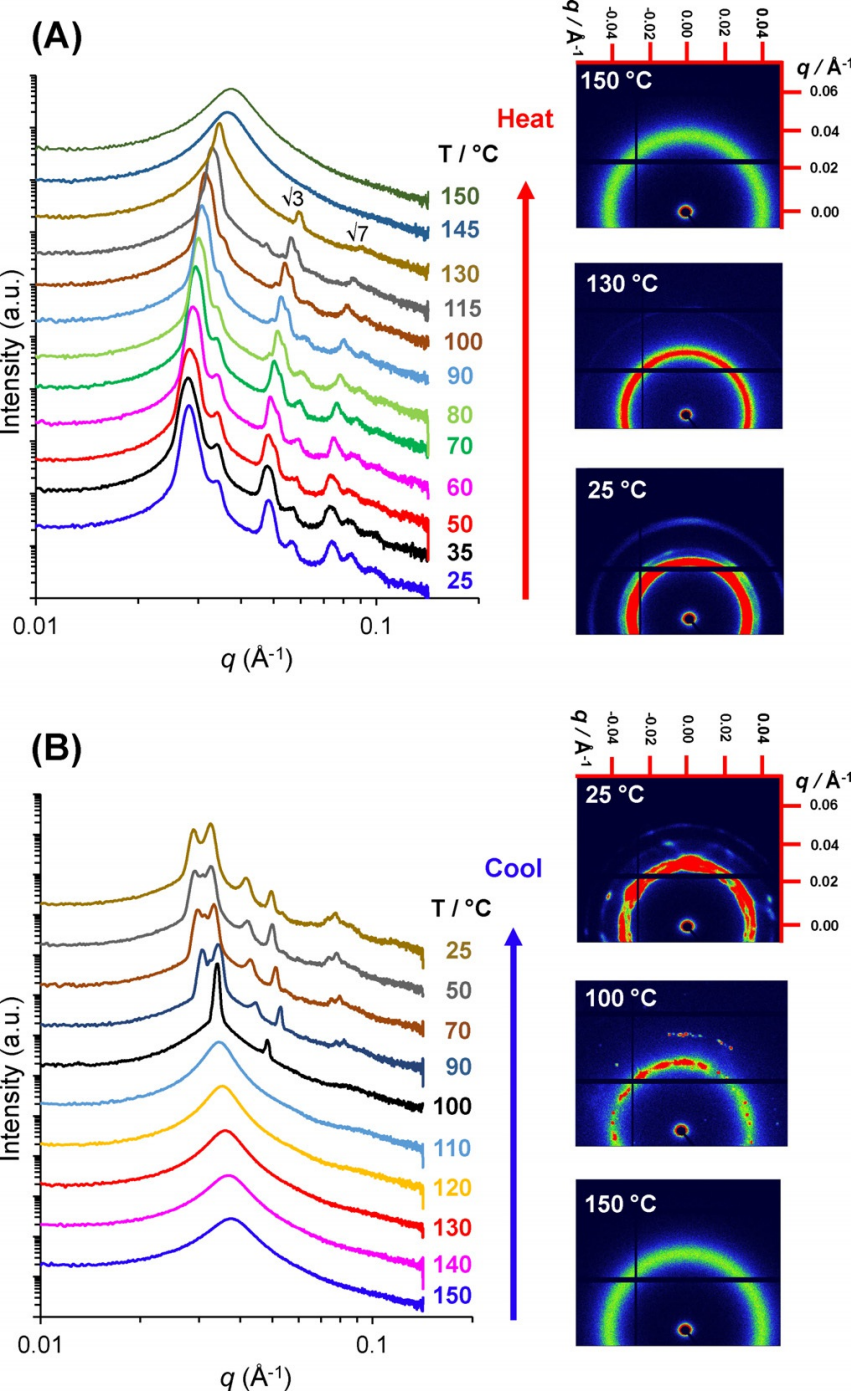
1D and 2D SAXS patterns recorded on A) heating a 40 % w/w dispersion of PhBD_80_–PBzMA_40_ copolymer in *n*‐dodecane from 25 °C to 150 °C and B) cooling the same dispersion from 150 °C to 25 °C. In each case, the temperature was increased or reduced at 5 °C intervals and the dispersion was equilibrated for 10 min at each temperature prior to data acquisition. 1D SAXS patterns are offset by an arbitrary multiplication factor to avoid overlap of the data.

**Variable‐temperature SAXS studies**. Various diblock copolymer nano‐objects prepared via RAFT dispersion polymerization have been reported to exhibit thermoresponsive behavior.[^14^, ^25^, ^28^, ^29^, ^30^, ^61^, ^70^, ^71^, ^72^] In some cases, such thermally induced transitions between copolymer morphologies can result in physical (de)gelation. To investigate the thermoresponsive behavior of the PhBD_80_–PBzMA_40_ nano‐objects in *n*‐dodecane, the as‐synthesized 40 % w/w dispersion was subjected to temperature‐dependent SAXS studies. At 25 °C, the initial dispersion mainly comprised peaks attributed to a hexagonal cylinder phase, although an unassigned peak on the high *q* side of the 10 reflection suggests coexistence of a second phase(s), see Figures 2 C and 3 A. On heating this concentrated dispersion up to 90 °C, the 20 reflection assigned to the hexagonal phase decreases in intensity, while a number of additional peaks are observed. Notably, the primary peak position shifts to higher *q* on heating (Figure 3 A). This temperature‐dependent reversible peak shift is likely to be related to the greater degree of solvation of the PBzMA core‐forming block at elevated temperature.^[61]^ In recent studies, such core solvation reduced the mean aggregation number of spherical nanoparticles comprising either PMMA or PBzMA cores, resulting in the formation of smaller nano‐objects at high temperature.[^73^, ^74^] In the present study, greater core solvation at the reaction temperature would account for the shorter inter‐micelle distances. Indeed, the diffraction peaks are observed at higher *q* values at 90 °C than at 25 °C. At 130 °C, SAXS studies reveal a diffuse isotropic peak that overlaps with a diffraction peak accompanied by two higher order Bragg peaks at *q*/q*=$\sqrt{3}$ and $\sqrt{7}$ (Figure 3 A). All the Bragg peaks disappear to leave just a single diffuse peak on further heating up to 145 °C, which indicates an ODT.[^59^, ^68^, ^75^] On cooling the resulting disordered phase from 150 °C to 100 °C, a series of Bragg spots reappear in the 2D SAXS patterns (Figure 3 B). The relative peak positions at *q*/q*=1 and $\sqrt{2}$ suggest that PhBD_80_–PBzMA_40_ forms a BCC phase at 100 °C, although there are too few peaks to enable an unambiguous assignment. However, further cooling produced qualitatively different SAXS patterns. At least seven peaks are observed at or below 90 °C, with peak positions corresponding to neither a BCC nor a HEX phase.

**Figure 4:**
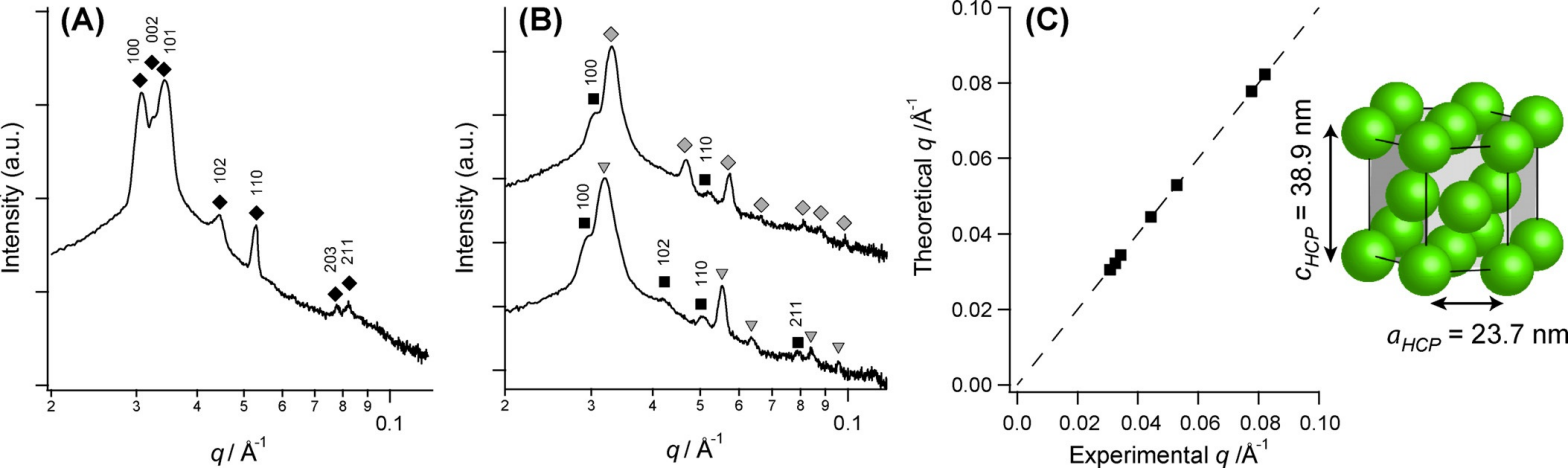
Assignment of the hexagonally close‐packed (HCP) sphere phase using the peak‐indexing protocols available in DataSqueeze 3.0 software.^[76]^ A) 1D SAXS pattern recorded at 90 °C during the cooling cycle of PhBD_80_–PBzMA_40_ nano‐objects from the disordered phase formed at 150 °C (Figure 3 B). B) Additional peaks (black squares) that are observed in coexistence with the BCC (upper pattern, gray diamonds) and hexagonal (lower pattern, gray triangles) phases can be assigned to Miller indices originating from the HCP sphere phase (see black squares). SAXS patterns are offset by an arbitrary multiplication factor to avoid overlap of the data. C) Correlation between the theoretical *q* values for Bragg peaks corresponding to an HCP phase and the experimental data determined from (A). The HCP phase comprises the unit cell shown in the inset, in which spheres are arranged within an HCP lattice with characteristic unit cell dimensions (*a*_HCP_ and *c*_HCP_).

The first three peaks are closely spaced (around 0.03 Å^−1^) and resemble the peak pattern expected for hexagonally close‐packed (HCP) spheres. This phase has been previously observed on numerous occasions for both block copolymers[^57^, ^77^] and surfactant lyotropic liquid crystals.[^78^, ^79^] Data analysis using peak‐indexing protocols available within DataSqueeze^®^ 3.0 software^[76]^ confirmed that all SAXS peaks observed on cooling from 90 °C (Figure 4 A) to 25 °C were consistent with an HCP phase (see Figure 4 C for experimental vs. theoretical peak indexing). The appearance of diffraction spots within the 2D SAXS pattern suggests that this concentrated diblock copolymer dispersion comprises large domains of HCP phase with differing orientations (Figure 3 B).^[57]^

The HCP phase has two characteristic unit cell parameters, describing the nearest neighbor spacing within one layer (*a*_HCP_), and the interlayer spacing between two layers with the same packing (*c*_HCP_), as shown in Figure 4 C. Both parameters increased on cooling from 90 °C (*a*_HCP_=23.7 nm and *c*_HCP_=38.9 nm) to 25 °C (*a*_HCP_=25.1 nm and *c*_HCP_=41.2 nm), while the *c*/*a* ratio remained constant at 1.64. For an ideal HCP phase, $c_{HCP}=2\sqrt{\left(\frac{2}{3}\right)}a_{HCP}$ , and hence *c*_HCP_/*a*_HCP_=1.63, which is in good agreement with the experimental data. Close inspection of the HCP phase diffraction patterns indicates anomalous peak broadening behavior, with the 110 peak being sharper than the 102 peak located at lower *q* (Figure 4 A). Since the 110 peak belongs to a family of peaks such that *h*−*k*=3 *n* (where *n* is an integer), this observation suggests the presence of stacking faults within the HCP structure. This is because broadening of the 110 reflection is independent of stacking faults while the 102 peak broadening is sensitive to such structural imperfections.^[80]^ Similar observations are often reported in the literature for close‐packed diblock copolymer spheres.^[81]^

The thermal annealing experiment suggests that HCP spheres is the thermodynamically preferred phase for a 40 % w/w PhBD_80_–PBzMA_40_ dispersion in *n*‐dodecane. Moreover, the phase transformations observed during thermal annealing suggest that the unassigned SAXS peaks observed during the PISA synthesis (see Figure 2 A,B) most likely belong to a coexisting HCP phase. In principle, the low *q* shoulder accompanying the first peak of the BCC and HEX phases observed at 0.029–0.031 Å^−1^ should correspond to the 100 reflection of the HCP phase that is observed at 0.030 Å^−1^ after thermal annealing (Figure 3 B, 25 °C), while the second unidentified peak at 0.039–0.041 Å^−1^ coincides with the 102 reflection. Applying the same HCP peak‐indexing protocol to the patterns recorded at ca. 72 min during in situ SAXS studies confirmed that every unidentified peak could be assigned to Miller indices of an HCP phase (Figure 4 B). This suggests that the HCP phase coexists with the BCC phase for reaction times between 72 and 76 min and remains thereafter with the HEX phase until the end of the polymerization.

Since the PhBD_80_–PBzMA_40_ diblock copolymer forms closed‐packed spheres under thermodynamic conditions that can coexist with other morphologies during copolymer synthesis, this suggests an alternative interpretation for the product that is formed after cooling the final copolymer dispersion to 25 °C (Figure 2 A). The diffraction pattern was initially attributed to a HEX cylinder phase (Figure 2 C, top). However, one unassigned peak at 0.033 Å^−1^ becomes more prominent for longer annealing times at elevated temperatures (Figure 3 A, see SAXS patterns between 35 and 60 °C) does not belong to the HEX phase. In fact, all but the principal diffraction peak can be assigned to a face‐centered cubic (FCC) structure formed by close‐packed spheres by using the 002 peak position (*q*=0.033 Å^−1^) as a reference for the FCC lattice period (*a*_FCC_=37.0 nm; Figure S5A). However, the most intense 111 peak of the FCC phase is slightly offset from the principal peak maximum, suggesting that if the observed pattern arose from an FCC phase then another phase must also be present. The copolymer chains self‐assemble to form worm‐like micelles (Figure 1 C,D), and all but one of the peaks (*q*=0.033 Å^−1^, assigned to the 002 reflection of FCC) can be assigned to the HEX cylinder phase (Figures 2 C and S5B). Hence it seems likely that both the FCC and HEX phases coexist in the final product after cooling to ambient temperature. This interpretation is supported by the SAXS patterns recorded at elevated temperatures (Figure S5C), where peak positions shift to reveal two sets of peaks that can be assigned to FCC and HEX phases.

Thus the sequence of phases formed during the PISA synthesis is BCC→BCC+HCP (with stacking faults)→HEX+HCP (with stacking faults), where the hexagonal cylinder phase (HEX) directly replaces the BCC phase. Such BCC→HEX morphological transitions are well‐documented for various block copolymer systems in the literature.[^47^, ^57^, ^66^, ^82^, ^83^, ^84^, ^85^] X‐ray diffraction patterns indicate that, on cooling the 40 % w/w copolymer dispersion to ambient temperature, the HCP phase (with its stacking faults) is transformed into an FCC structure of close‐packed spheres. Thus, the final copolymer dispersion at 25 °C is composed of HEX and FCC (possibly with associated stacking faults) formed by worm‐like and spherical micelles, respectively.

**Reaction phase diagram**. In addition to the observed phase transitions, the gradual shift in the Bragg peaks to lower *q* during the BzMA polymerization indicates progressively larger domain spacings. More detailed analysis of the SAXS patterns enables the varying dimensions of these ordered phases to be assessed. In the early stages of the polymerization, the system comprised a disordered array of micelles which most likely possess a pseudo‐spherical morphology. The mean distance between nearest neighbor micelles (*D*_DIS_) can be estimated from the structure factor peak maximum (q*) using the simple relationship $D_{DIS}=\;$ 2π/q*. SAXS patterns recorded in situ during the polymerization indicate the presence of a BCC phase between 60 and 74 min, with the unit cell size increasing from 24.1 to 26.6 nm during this interval. From these dimensions, the nearest neighbor center‐to‐center distance (*D*_BCC_) can be calculated from the unit cell size using the equation $D_{BCC}=\;\frac{\sqrt{3}a_{BCC}}{2}$ .^[57]^ Similar structural parameters can be calculated for the HEX phase, which becomes the dominant phase within 80 min. The mean center‐to‐center distance for nearest neighbor cylinders (*D*_HEX_) can be calculated using ${a_{HEX}=D}_{HEX}=\;\frac{2d_{10}}{\sqrt{3}}$ .^[57]^ Finally, within the coexisting HCP phase, the center‐to‐center distance (*D*_HCP_) for neighboring spherical micelles is simply the unit cell parameter *a*_HCP_, i.e., $D_{HCP}=a_{HCP}$ .^[57]^

Mean inter‐micelle distances increase from 20.9 to 23.1 nm between 60 and 74 min. This greater inter‐separation distance between neighboring micelles is the result of the mean micelle core radius increasing by approximately 1.1 nm as the core‐forming PBzMA block grows longer. There is also a modest increase in the mean inter‐cylinder separation distance, from 22.2 nm after 80 min to 23.2 nm at the end of the BzMA polymerization.

**Figure 5:**
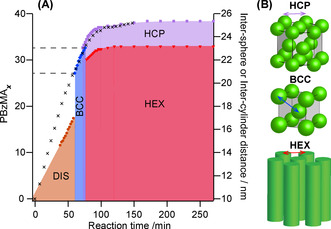
A) Reaction phase map recorded during the PISA synthesis of PhBD_80_–PBzMA_40_ diblock copolymer nano‐objects at 40 % w/w solids in *n*‐dodecane. Colored symbols denote domain spacings within different phases calculated from time‐resolved SAXS data, while black crosses indicate the mean degree of polymerization (*x*) of the insoluble PBzMA block calculated from in situ ^1^H NMR studies. The two dashed lines shown on the left indicate the approximate time points at which the disorder–order and order–order phase transitions occur. B) Schematic cartoons illustrate the inter‐sphere distances for the hexagonally close‐packed (HCP) and body‐centered cubic (BCC) phases and the inter‐cylinder distance for hexagonally packed cylinders (HEX). The green spheres and cylinders represent the PBzMA cores of nano‐objects that form structured arrangements within a continuous phase comprising PhBD_80_ chains and *n*‐dodecane.

From the SAXS patterns recorded over the course of the BzMA polymerization, the mean distance between spherical micelle cores or cylinder cores can be plotted as a function of time (Figure 5, right axis). To enable assignment of precise diblock compositions at specific time intervals, kinetic studies were performed targeting a 40 % w/w dispersion of PhBD_80_–PBzMA_40_ worms in *n*‐dodecane at 90 °C using in situ ^1^H NMR spectroscopy. The resulting BzMA conversion vs. time curve (Figure S5) was used to calculate the instantaneous mean degree of polymerization (DP) for the growing PBzMA block (Figure 5, left axis) and plotted alongside the domain spacing data. It is noteworthy that a constant domain spacing was observed after around 150 min for both the cylinder and the HCP phases. This is consistent with the conversion vs. time curve obtained from ^1^H NMR studies, which confirmed that 95 % BzMA conversion was achieved within this timescale (Figure S5). Hence the instantaneous mean DP of the PBzMA block can be calculated at any given time point during the in situ SAXS experiments, which enables the morphological development to be understood in terms of the diblock composition.

The reaction phase map indicates that, although the *d*‐spacing increases over the course of the reaction, large jumps occur during the phase transitions. For example, when passing from the disordered to the ordered micellar phase, the mean inter‐micelle distance increases significantly from approximately 17 to 21 nm. This onset of long‐range order is considered to be a transition between weakly and strongly segregated states, and it occurs when the mean PBzMA DP is approximately 27.^[86]^ This phase change is accompanied by significant changes in the conformation of each block. More specifically, as the enthalpic interaction between the two blocks increases, they become more perturbed from their Gaussian coil conformations.^[87]^ Simultaneous stretching of both the core‐forming and corona‐forming chains, which is required to minimize their interfacial contact area, results in a dramatic increase in the mean separation distance between micelle cores. After approximately 72 min, which corresponds to an instantaneous diblock composition of PhBD_80_–PBzMA_31_, an HCP phase coexists with the BCC phase with virtually the same inter‐sphere spacing being determined for these two phases.

However, when the BCC phase evolves to form a HEX phase (at an intermediate PhBD_80_–PBzMA_33_ composition), the mean inter‐cylinder distance within the 2D hexagonal phase is significantly smaller—by up to 2 nm—than the inter‐sphere distance within the co‐existing HCP phase (and indeed the precursor BCC phase). Park et al. reported similar observations for PS–PI diblock copolymers dissolved in diethyl phthalate (a selective solvent for the polystyrene block) during a thermally induced transition from a BCC/HCP phase to a hexagonal lyotropic phase.^[57]^ These findings suggest that the spherical micelles fuse to form cylinders along the BCC ⟨111⟩ direction during the BCC‐to‐hexagonal cylinder phase transition.^[65]^ If this is correct, then the mean distance between columns of spheres that fuse along the ⟨111⟩ directions should be equivalent to the inter‐cylinder distance. The value calculated for the BCC phase just prior to the phase transition (22.0 nm) agrees rather well with the inter‐cylinder distance of 22.2 nm determined after the transition. The observed transformation of spheres into worms during the PISA synthesis is favored because of the crystallographic relationship between BCC and HEX phases. However, spheres packed in another (e.g. HCP) phase maintain their particle morphology until the BzMA polymerization is complete. As far as we are aware, this is the first demonstration that such a phase transition can be induced during the synthesis of diblock copolymer chains. In this context, it is noteworthy that Hillmyer and co‐workers reported using RAFT polymerization in the absence of solvent to drive microphase separation in the solid state, but the resulting diblock copolymers did not form such highly ordered structures.[^88^, ^89^, ^90^]

## Conclusion

Time‐resolved SAXS has been used to monitor the evolution in copolymer morphology that occurs during the PISA synthesis of PhBD_80_–PBzMA_40_ diblock copolymer worms at 90 °C in *n*‐dodecane when targeting 40 % w/w solids. As the structure‐directing PBzMA block grows during this PISA synthesis, there is a gradual evolution from molecularly dissolved copolymer chains to spheres to close‐packed spheres (BCC/HCP phases) to a final mixture of HEX and HCP phases (where HEX denotes hexagonally packed cylinders—or partially aligned worms—and is the major phase). SAXS analysis suggests that this HEX phase is generated via sphere–sphere fusion within the BCC phase. To the best of our knowledge, this is the first time that any long‐range order has been observed for block copolymer nano‐objects during their PISA synthesis. It is emphasized that this is achieved using an amorphous core‐forming block, rather than a (liquid) crystalline block.^[49]^ Serial dilution of the HEX/HCP phase leads to the formation of a disordered phase comprising mainly non‐interacting worms at 1.0 % w/w. Thermal annealing of the as‐synthesized 40 % w/w PhBD_80_–PBzMA_40_ dispersion induces a cylinder‐to‐sphere transition at 150 °C to produce a disordered sphere phase. On cooling to 25 °C, the spheres form an HCP lattice, just like the minor phase that co‐existed with the HEX phase during PISA. The observation of a lamellar phase for the near‐symmetric PhBD_80_–PBzMA_40_ diblock copolymer in the solid state indicates that selective swelling of the PhBD_80_ block by *n*‐dodecane results in myriad ordered morphologies that are generated during the BzMA polymerization and/or upon thermal annealing. Finally, the basic principles of block copolymer self‐assembly suggest that our observations should also apply to other PISA formulations.[^11^, ^46^, ^48^] However, further work is required to confirm such generic behavior.

## Conflict of interest

The authors declare no conflict of interest.

## Supporting information

As a service to our authors and readers, this journal provides supporting information supplied by the authors. Such materials are peer reviewed and may be re‐organized for online delivery, but are not copy‐edited or typeset. Technical support issues arising from supporting information (other than missing files) should be addressed to the authors.

Supplementary
