# Supplementary material for: Small‐Angle X‐Ray Scattering Studies of Block Copolymer Nano‐Objects: Formation of Ordered Phases in Concentrated Solution During Polymerization‐Induced Self‐Assembly
Source: Angew Chem Int Ed Engl. 2021 May 1;60(23):12955–63. doi: 10.1002/anie.202101851 (PMC8252599; doi:10.1002/anie.202101851)
Supplement: Supplementary file 1 — Supplementary [file ANIE-60-12955-s001.pdf]

Angewandte Chemie
Eine Zeitschrift der Gesellschaft Deutscher Chemiker

Supporting Information

Small-Angle X-Ray Scattering Studies of Block Copolymer Nano-Objects: Formation of Ordered Phases in Concentrated Solution During Polymerization-Induced Self-Assembly

Matthew J. Rymaruk[+], Cate T. O'Brien[+], Csilla György, Bastien Darmau, James Jennings, Oleksandr O. Mykhaylyk,* and Steven P. Armes**

anie_202101851_sm_miscellaneous_information.pdf

Summary of Supporting Information

Contents	Page

Experimental Details S2

Figure S1: GPC analysis of the $PhBD_{80}$ precursor and $PhBD_{80}$-$PBzMA_{40}$ diblock copolymer S5

Figure S2: ^1H NMR spectrum for $PhBD_{80}$-$PBzMA_{40}$ diblock copolymer S6

Figure S3: *In situ* SAXS data from the first 58 mins of $PhBD_{80}$-$PBzMA_{40}$ synthesis S7

Figure S4: Temperature-dependent SAXS data of $PhBD_{80}$-$PBzMA_{40}$ in the solid state S8

Figure S5: SAXS analysis demonstrating the coexistence of FCC with HEX phase S9

Figure S6: *In situ* ^1H NMR study of $PhBD_{80}$-$PBzMA_{40}$ synthesis S10

References S10

Experimental section

Materials. Monohydroxy-capped hydrogenated polybutadiene (M_n = 4 500 g mol^{-1}, Đ = 1.09, comprising 61 mol% 1,4-polybutadiene and 39 mol% 1,2-polybutadiene) was kindly donated by Kraton Polymers LLC. (Houston, Texas, USA) and used as received. 4-(Dimethylamino)pyridine (DMAP), *N,N'*-dicyclohexylcarbodiimide (DCC), dichloromethane, benzyl methacrylate, *n*-dodecane, methanol, tetrahydrofuran (THF), 3,5-di-tert-4-butylhydroxytoluene (BHT) and triethylamine were purchased from Sigma Aldrich (UK). Trigonox 21S initiator was purchased from AkzoNobel (The Netherlands). Deuterated chloroform ($CDCl_3$) and deuterated dichloromethane (CD_2Cl_2) were purchased from Goss Scientific (UK). 4-Cyano-4-(2-phenylethane sulfanyl- thiocarbonyl)sulfanylpentanoic acid (PETTC) RAFT agent was prepared in-house according to a previously reported protocol.[1,2] Borosilicate glass capillaries (2 mm diameter) used as sample holders for SAXS measurements were purchased from WJM-Glass (Germany).

Methods

Synthesis of the trithiocarbonate-capped hydrogenated polybutadiene precursor. Monohydroxy-capped hydrogenated polybutadiene (PhBD; 9.00 g, 2.00 mmol) and the carboxylic acid-functionalized PETTC RAFT agent (1.36 g, 4.00 mmol) were weighed out into a round-bottomed flask containing a magnetic stirrer bar and dried in a vacuum oven overnight. The flask was equipped with a reflux condenser, evacuated under vacuum and refilled with nitrogen gas. Anhydrous dichloromethane (50 mL) was then added via syringe, along with DMAP (49.0 mg, 0.40 mmol, dissolved in 1.0 mL anhydrous dichloromethane prior to addition). The resulting mixture was then cooled to 0 °C for 30 min by immersing the flask in an ice bath, before DCC (1.0 g, 5.0 mmol, dissolved in 5.0 mL anhydrous dichloromethane prior to addition) was added dropwise over 20 min. The reaction mixture was then maintained at 0 °C for 60 min before being heated to reflux for 40 h. The reaction mixture was then cooled, placed in a freezer overnight, and then filtered to remove the dicyclohexylurea precipitate. Finally, dichloromethane was removed from the crude reaction mixture under reduced pressure, prior to purification by precipitation into a ten-fold excess of methanol (three times). ^{1}H NMR spectroscopy studies indicated that 96% of the terminal hydroxy groups had been esterified, (the integrated proton signals assigned to the PhBD backbone were compared to the integrated signal assigned to the five aromatic protons associated with the phenyl end-group on the PETTC RAFT agent). UV spectroscopy studies conducted in dichloromethane indicated a mean degree of esterification of 96% by end-group analysis assuming a molar extinction coefficient of 10400 L mol^{-1} cm^{-1} for the trithiocarbonate end-group.

Synthesis of $PhBD_{80}$-$PBzMA_{40}$ worms by RAFT dispersion polymerization of BzMA in *n*-dodecane. A typical synthesis of $PhBD_{80}$-$PBzMA_{40}$ worms at 40% w/w solids in *n*-dodecane was conducted as follows (**Figure 1**): PhBD RAFT agent (2.0 g, 0.46 mmol), BzMA (3.20 g, 17.7 mmol, target DP = 40 assuming a mean degree of esterification of 96% for this PhBD precursor) and *n*-dodecane (7.72 g) were weighed into a 10 mL glass vial equipped with a magnetic stirrer. T21s initiator (20.0 mg, 92.6 μmol, [PhBD]/[T21s] molar ratio = 5.0; 222 μL of a 10% v/v solution in *n*-dodecane) was added to this solution at ambient temperature. The resulting mixture was then purged with nitrogen, sealed, and placed in a pre-heated oil bath set at 90 °C for 5 h. After cooling to ambient temperature, the final dispersion was obtained as a yellow free-standing gel. ^{1}H NMR spectroscopy studies ($CDCl_3$) confirmed a BzMA conversion of 99%, while THF GPC analysis indicated an M_n of 14 300 g mol^{-1} and an M_w/M_n of 1.17.

SAXS Instrumentation. SAXS patterns were recorded using a Xeuss 2.0 laboratory beamline (Xenocs, Grenoble, France) equipped with a Pilatus 1M detector (Dectris, Baden, Switzerland)

and a liquid gallium MetalJet X-ray source (λ = 1.34 Å) (Excillum, Kista, Sweden). Typically, patterns were collected over a q range of 0.003 to 0.16 $Å^{-1}$. The length of the scattering vector, q, is given by $q = (4\pi/\lambda) \sin \theta$, where θ is half the scattering angle. A heating/cooling capillary stage (Linkam, Tadworth, UK) was used to provide temperature control for the *in-situ* polymerization and variable temperature studies. For samples analysed at 1 % w/w copolymer concentration, background subtraction and normalization were performed. Because of a strong scattering signal produced by samples with 5 – 40 % w/w copolymer concentrations (which was significantly higher than background scattering), no background scattering subtraction was performed for their SAXS patterns. Highly viscous concentrated dispersions of diblock copolymer worms were studied using a bespoke brass 'gel cell' equipped with Kapton windows (16 mm diameter, 0.25 mm thickness). A 1 mm thick polypropylene spacer was employed to ensure a consistent sample thickness. For further details, see the Supporting Information.

In situ SAXS experiments during the PISA synthesis of $PhBD_{80}$-$PBzMA_{40}$ worms at 40 w/w %. In situ SAXS studies were conducted during the PISA synthesis of $PhBD_{80}$-$PBzMA_{40}$ worms according to the following protocol (**Figure 1**). First, trithiocarbonate-capped hydrogenated polybutadiene ($PhBD_{80}$) was prepared by esterification of a monohydroxy-capped hydrogenated polybutadiene precursor that had been prepared by anionic polymerization, as been described previously.[3] $PhBD_{80}$ RAFT agent (0.30 g, 62.0 µmol), BzMA monomer (0.42 g, 2.39 mmol, target DP = 40 assuming a mean degree of esterification of 96% for this $PhBD_{80}$ precursor) and *n*-dodecane (1.08 g) were weighed into a 10 mL glass vial equipped with a magnetic stirrer. T21s initiator (2.70 mg, 12.4 µmol, $[PhBD_{80}]/[T21s]$ molar ratio = 5.0; 29 µL of a 10% v/v solution in *n*-dodecane) was added to this solution at 20 °C. The resulting mixture was then sealed with a rubber septum and purged with nitrogen gas for 20 min. Finally, approximately 0.10 mL of this solution was removed via syringe and transferred to a glass capillary of 2 mm diameter. While transferring this reaction mixture, a gentle stream of nitrogen gas was passed over the opening of the capillary to exclude air. The capillary was then sealed using epoxy resin and transferred to a Linkam heating/cooling stage. The capillary was then heated to 90 °C and each SAXS pattern was acquired for 2 min with each subsequent pattern being acquired immediately thereafter for 270 min (i.e., 135 patterns were recorded over the course of the BzMA polymerization). GPC analysis indicated an M_n of 14,600 g mol^{-1} and an M_w/M_n of 1.10 (**Figure S1**) while ^{1}H NMR studies confirmed a final BzMA conversion of 99% after 16 h at 90 °C (**Figure S2**).

A further batch of $PhBD_{80}$-$PBzMA_{40}$ worms was also prepared on a 10-gram scale at 40% w/w solids in *n*-dodecane in a glass sample vial with continuous magnetic stirring. This dispersion was then serially diluted by adding the required amount of *n*-dodecane to a vial and placing on a roller mill for 48 h to ensure homogenization. Unfortunately, 10-40 % w/w copolymer dispersions were too viscous to transfer into a 2.0 mm capillary cell. Thus such dispersions were placed between two Kapton disks prior to analysis. The SAXS pattern observed after cooling the 40 % w/w $PhBD_{80}$-$PBzMA_{40}$ dispersion prepared in the glass vial to 25 °C (Figure 2D, uppermost trace) was almost identical to that recorded for the same PISA synthesis performed in a 2.0 mm capillary cell (Figure 2A, uppermost trace). This indicates that lyotropic order is not merely an artefact of capillary confinement and can be obtained in the presence or absence of shear.

Variable temperature SAXS studies of a 40% w/w dispersion of $PhBD_{80}$-$PBzMA_{40}$ worms. The PISA formulation used to prepare a 40% w/w dispersion of $PhBD_{80}$-$PBzMA_{40}$ worms was also used for variable temperature SAXS studies. Again, approximately 0.10 mL of this solution was removed via syringe and transferred to a glass capillary of 2 mm diameter, which was then sealed using epoxy resin and placed in a pre-heated oil bath set at 90 °C for

approximately 16 h. The capillary was then removed from the oil bath, allowed to cool to ambient temperature, and gently wiped with ethanol and acetone to remove any residual oil prior to SAXS analysis. The capillary was then heated from 25 °C to 150 °C and cooled to 25 °C in 5 °C increments using the same Linkam heating/cooling stage. In this experiment, 10 min was allowed for thermal equilibration at each temperature step before SAXS pattern was acquired for 5 min.

^{1}H NMR spectroscopy. ^{1}H NMR spectra were recorded in either CD_2Cl_2 or $CDCl_3$ using a Bruker AV1-400 MHz spectrometer. Typically, 64 scans were averaged per spectrum.

Gel permeation chromatography. Molecular weight distributions were determined using a GPC set-up operating at 30 °C that comprised two Polymer Laboratories PL gel 5 μm Mixed C columns, a LC20AD ramped isocratic pump, THF eluent and a WellChrom K-2301 refractive index detector operating at 950 ± 30 nm. The mobile phase contained 2.0 % v/v triethylamine and 0.05 % w/v 3,5-di-tert-4-butylhydroxytoluene (BHT) and the flow rate was 1.0 ml min^{-1}. A series of ten near-monodisperse poly(methyl methacrylate) standards (M_n = 1 280 to 330 000 g mol^{-1}) were used for calibration. Chromatograms were analyzed using Varian Cirrus GPC software.

Transmission electron microscopy. Transmission electron microscopy (TEM) studies were conducted using a FEI Tecnai G2 spirit instrument operating at 80 kV and equipped with a Gatan 1k CCD camera. Copper TEM grids were surface-coated with a thin film of amorphous carbon using an in-house coating protocol. A single droplet of a 0.20 % w/w copolymer dispersion was loaded onto a carbon-coated grid and allowed to dry at 20 °C. Prior to imaging, each grid was exposed to ruthenium(IV) vapor for 7 min at 20 °C in order to achieve sufficient contrast. This heavy metal staining agent was prepared by adding ruthenium(II) oxide (0.30 g) to water (50 g) to form a slurry. Then sodium periodate (2.0 g) was added to the stirred solution and a yellow solution of ruthenium(IV) oxide was formed within 1 min at 20 °C.[4]

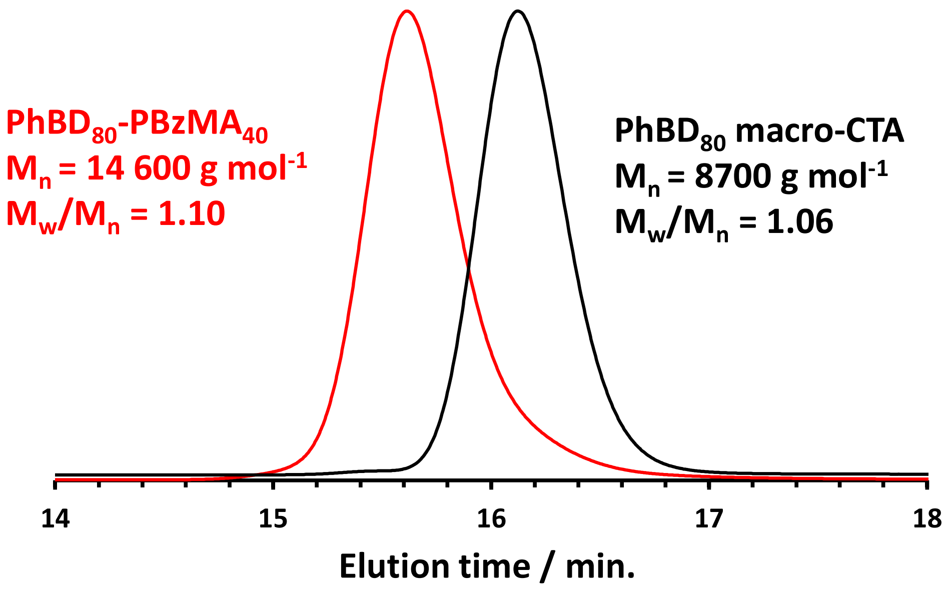

Figure S1. Gel permeation chromatograms (THF eluent, RI detection) recorded for the $PhBD_{80}$ precursor (black curve) and the final $PhBD_{80}$-$PBzMA_{40}$ diblock copolymer (red curve). Molecular weight data were calculated after calibration using a series of low-dispersity PMMA standards.

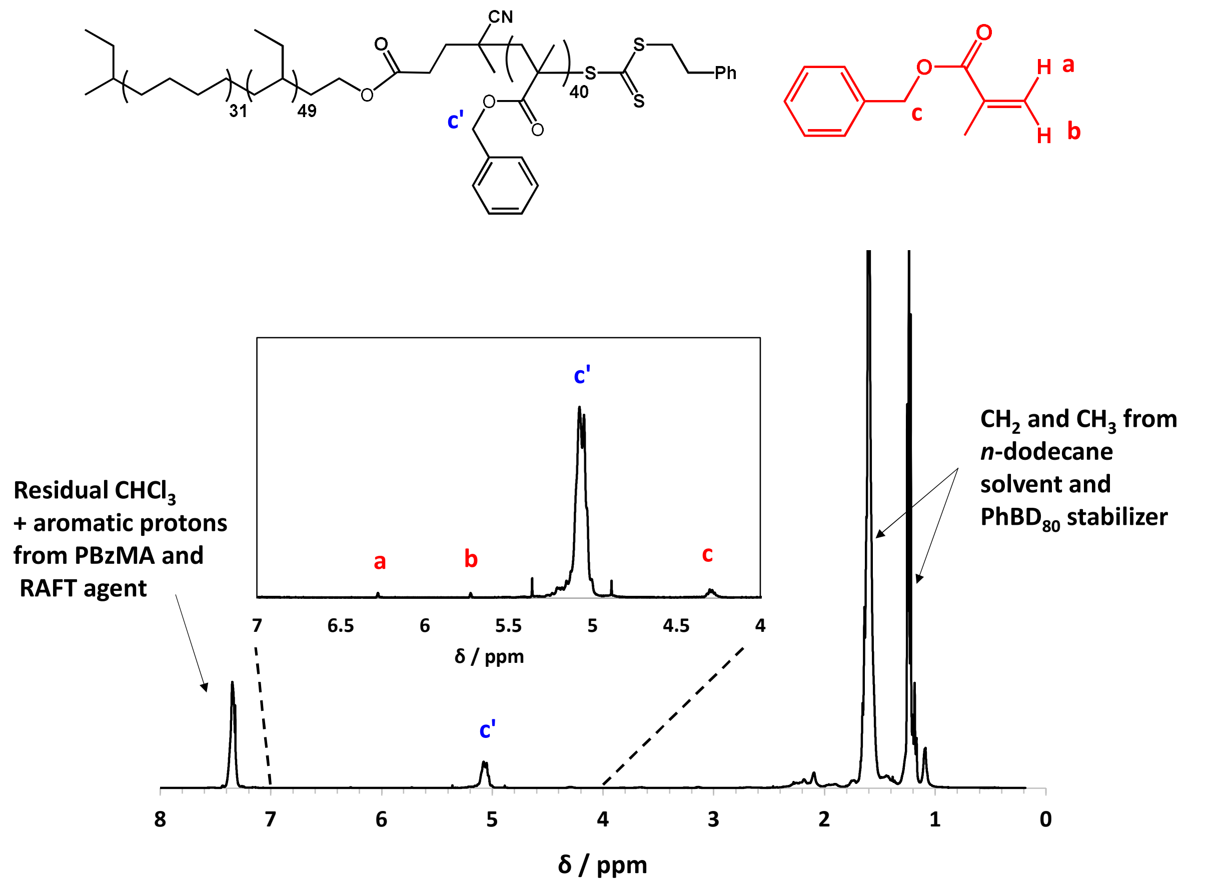

Figure S2. ^{1}H NMR spectrum recorded in $CDCl_3$ for the $PhBD_{80}$-$PBzMA_{40}$ diblock copolymer initially prepared as a 40% w/w dispersion of diblock copolymer worms in *n*-dodecane. The BzMA monomer conversion was determined to be 99% as follows: the oxymethylene proton signals assigned to the BzMA monomer (c) and PBzMA block (c') were integrated between 4.3 ppm and 5.5 ppm. The vinyl signals assigned to the BzMA monomer at 6.2 ppm and 5.6 ppm (see a and b, respectively) were then compared to these c and c' signals.

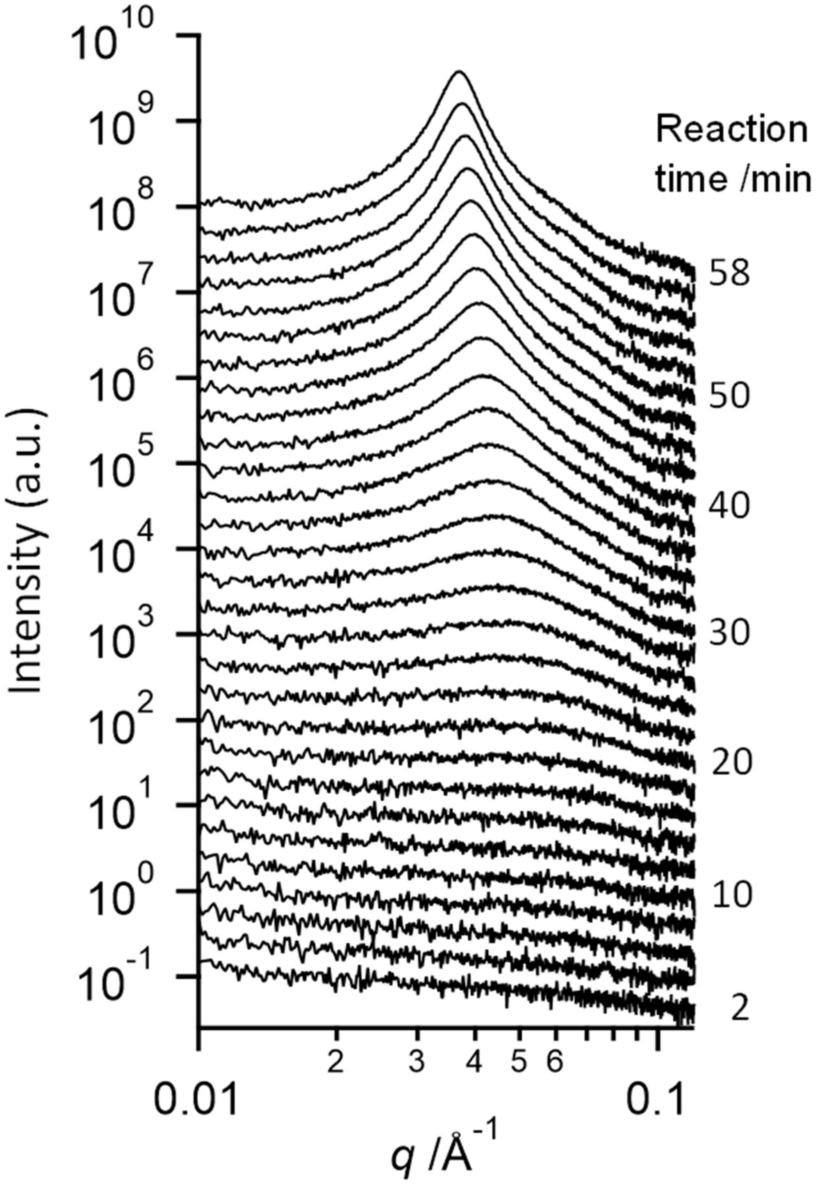

Figure S3. SAXS data recorded during the first 58 min of the RAFT dispersion polymerization of BzMA to form $PhBD_{80}$-$PBzMA_{40}$ worms at 40% w/w solids in *n*-dodecane. The sharper peaks observed at higher BzMA conversion indicates greater correlation between scattering objects, while the shift towards lower *q* indicates that the mean separation distance between scattering objects increases during this polymerization. SAXS patterns are offset by an arbitrary multiplication factor to avoid overlap of the data.

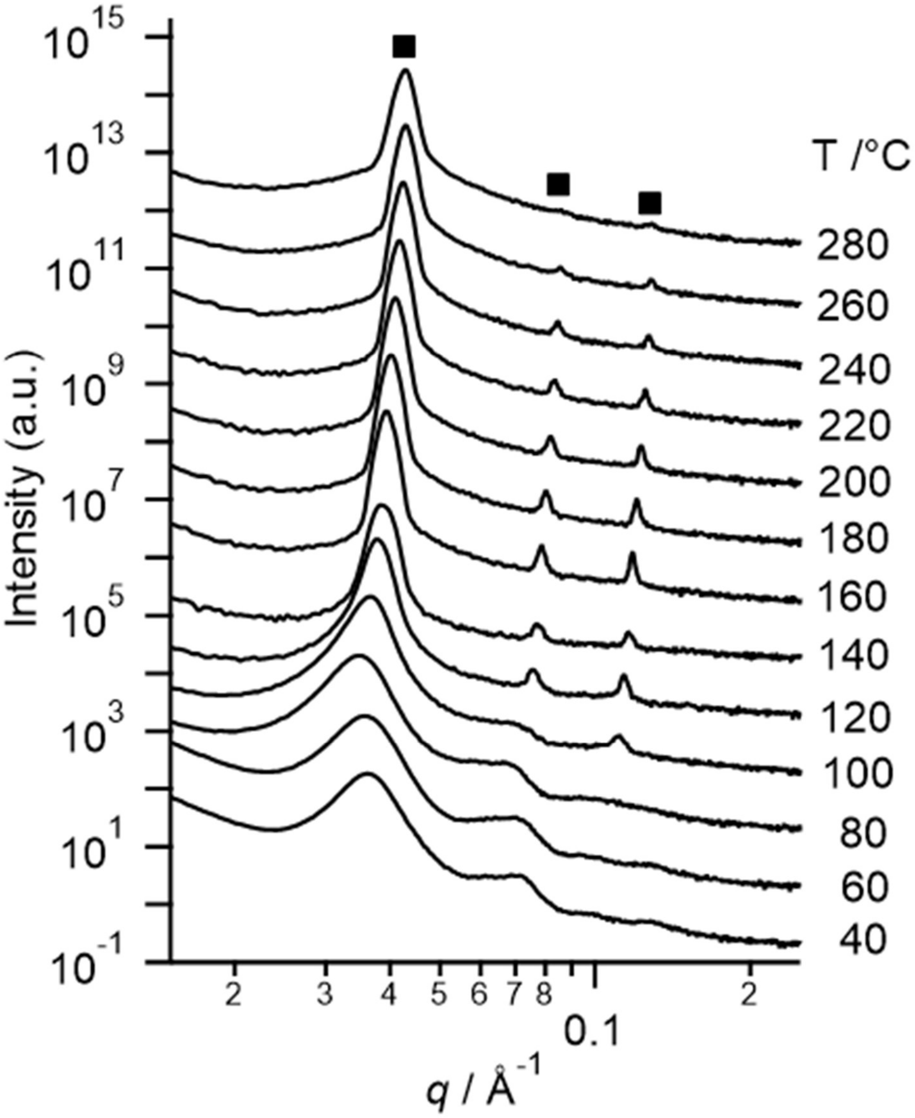

Figure S4. SAXS patterns were recorded during thermal annealing of PhBD$_{80}$-PBzMA$_{40}$ in the solid state after isolation by precipitating the as-synthesized 40% w/w copolymer dispersion in n-dodecane into excess ethanol, followed by drying to remove residual solvent. The appearance of a lamellar phase on heating above 100 °C is indicated by the emergence of sharp, equally-spaced Bragg peaks (labelled with black squares in the top trace) at q/q^* = 1, 2 and 3. SAXS patterns are offset by an arbitrary multiplication factor to avoid overlap of the data.

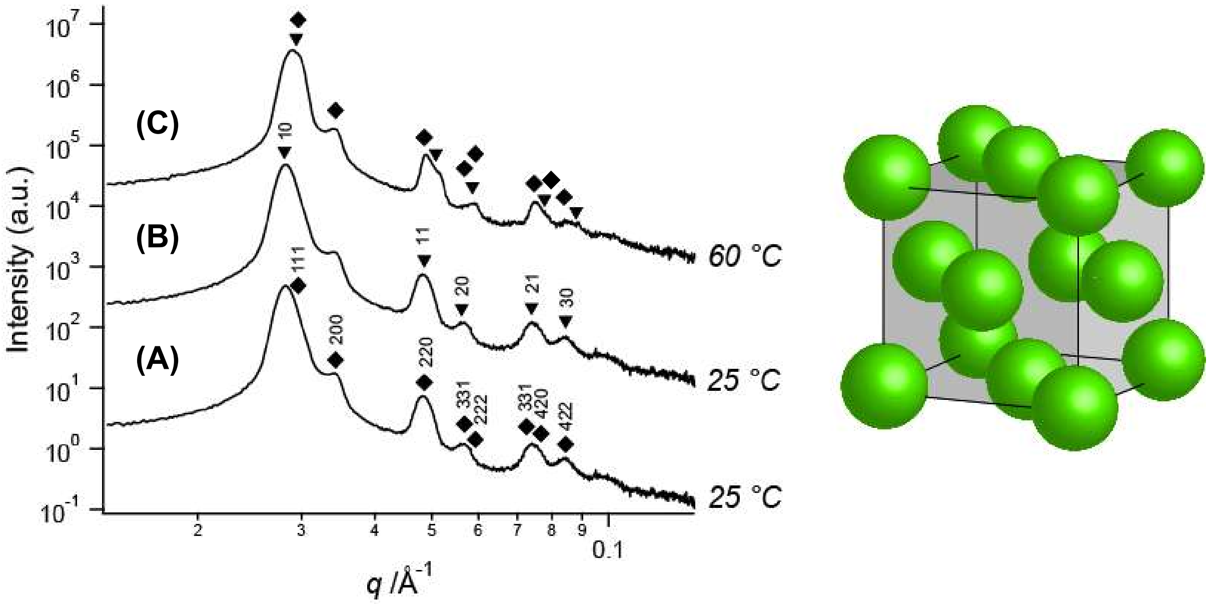

Figure S5. Assignment of the face-centred cubic (FCC) sphere phase using the peak-fitting protocol available in DataSqueeze 3.0 software.[5] 1D SAXS patterns (also shown in **Figure 3A**) obtained on cooling as-synthesised $PhBD_{80}$-$PBzMA_{40}$ nano-objects at 40% w/w to 25 °C (A and B), and upon reheating to 60 °C (C). Diffraction peaks corresponding to FCC phase are labeled by Miller indices, *hkl*, (A) and indicated by diamonds. For the same scattering pattern, diffraction peaks corresponding to HEX phase are labeled by Miller indices, *hk*, (B) and indicated by triangles. At 60 °C, peaks arising from HEX and FCC phases become separated, confirming the coexistence of the two phases during the heating cycle. SAXS patterns are offset by an arbitrary multiplication factor to avoid overlap of the data.

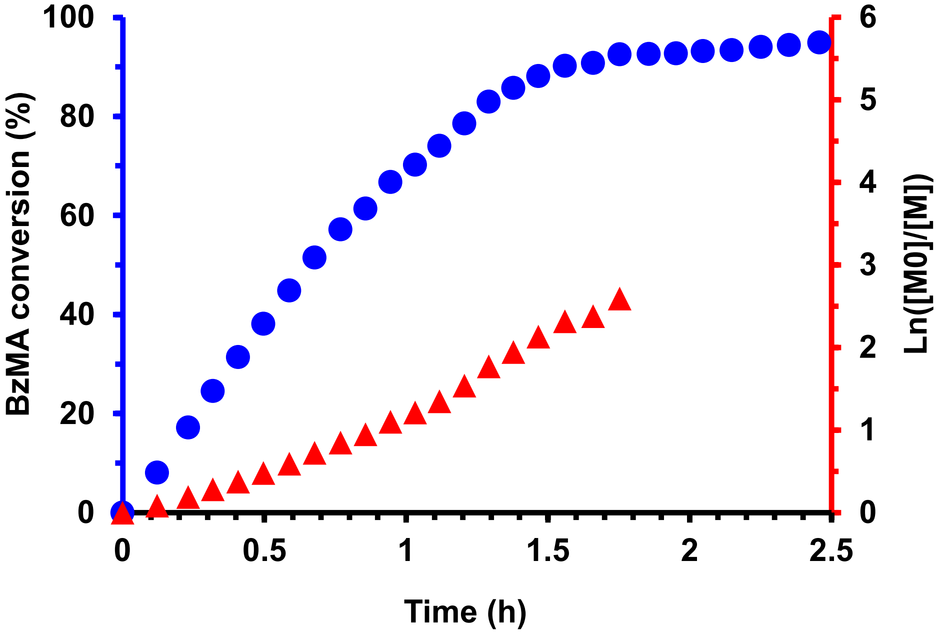

Figure S6. Conversion vs. time curve and the corresponding semi-logarithmic plot obtained by *in situ* ^{1}H NMR spectroscopy studies of the RAFT dispersion polymerization of BzMA at 90 °C targeting PhBD$_{80}$-PBzMA$_{40}$ worms at 40% w/w solids in *n*-dodecane.

References

(1) Semsarilar, M.; Ladmiral, V.; Blanazs, A.; Armes, S. P. Anionic Polyelectrolyte-Stabilized Nanoparticles via RAFT Aqueous Dispersion Polymerization. *Langmuir* **2012**, *28*, 914–922.

(2) Rymaruk, M. J.; Thompson, K. L.; Derry, M. J.; Warren, N. J.; Ratcliffe, L. P. D.; Williams, C. N.; Brown, S. L.; Armes, S. P. Bespoke Contrast-Matched Diblock Copolymer Nanoparticles Enable the Rational Design of Highly Transparent Pickering Double Emulsions. *Nanoscale* **2016**, *8*, 14497–14506.

(3) Darmau, B.; Rymaruk, M. J.; Warren, N. J.; Bening, R.; Armes, S. P. RAFT Dispersion Polymerization of Benzyl Methacrylate in Non-Polar Media Using Hydrogenated Polybutadiene as a Steric Stabilizer Block. *Polym. Chem.* **2020**, *11*, 7533–7541.

(4) Trent, J. S.; Scheinbeim, J. I.; Couchman, P. R. Ruthenium Tetraoxide Staining of Polymers for Electron Microscopy. *Macromolecules* **1983**, *16*, 589–598.

(5) https://www.physics.upenn.edu/~heiney/datasqueeze/.
